# Association between meat consumption and cancer mortality in Korean adults

**DOI:** 10.3389/fnut.2026.1811743

**Published:** 2026-05-04

**Authors:** In Sun Ryou, Hyun Jeong Cho, Yeo Ju Sohn, Jung Eun Lee, Minseon Park

**Affiliations:** 1 Department of Family Medicine, Ewha Womans University Seoul Hospital, Ewha Womans University College of Medicine, Seoul, Republic of Korea; 2 Department of Food and Nutrition, College of Human Ecology, Seoul National University, Seoul, Republic of Korea; 3 Department of Family Medicine, Seoul National University Hospital, Seoul National University College of Medicine, Seoul, Republic of Korea

**Keywords:** breast cancer, cancer mortality, meat consumption, nutritional epidemiology, organ meat, pancreatic cancer

## Abstract

**Background:**

Evidence regarding the association between meat consumption and cancer mortality remains inconsistent. Most previous studies have focused on total or red meat intake and cancer incidence, with limited data on cancer-specific mortality in Asian populations. Accordingly, this study aimed to investigate the associations between the consumption of various meat types and site-specific cancer mortality among Korean adults.

**Methods:**

We analyzed data from the Korean Genome and Epidemiology Study (KoGES), a large population-based cohort in South Korea. Meat intake was assessed using validated food frequency questionnaires and categorized into total meat and specific subtypes. Cancer-specific mortality was ascertained through national death registry linkage. Hazard ratios and 95% confidence intervals were estimated using Cox proportional hazards models adjusted for major demographic, lifestyle, and dietary factors, with sex-specific and stratified analyses.

**Results:**

Total meat consumption was not significantly associated with overall cancer mortality in either men or women. However, sex-specific associations were observed according to meat subtype. Among men, higher red meat consumption was inversely associated with gastric cancer mortality. Among women, higher organ meat consumption was associated with increased pancreatic and breast cancer mortality, with stronger associations observed among women with lower body mass index, older age, and never-smoking status.

**Conclusion:**

In this study, total meat intake was not associated with overall cancer mortality, whereas cancer-specific associations differed by meat subtype and sex. These findings suggest that detailed characterization of meat consumption may be important for understanding cancer mortality patterns in Asian populations.

## 1 Introduction

Meat consumption has increased in many Asian populations (1). A recent systematic review and meta-analysis reported that meat consumption increased by 21.1 g/day per decade in Southeast Asia and by 12.8 g/day per decade in East Asia over the past 70 years (2). In South Korea, per-capita meat consumption in 2018 was approximately twice that observed in 1998, indicating a substantial increase over the past two decades (3).

Meat consumption is a well-recognized modifiable dietary factor in cancer risk. Long-term consumption of processed meat (e.g., sausages, bacon, ham, beef jerky, corned beef, and other smoked, salted, fermented, or cured meats) and red meats (e.g., beef, pork, and lamb) has been consistently associated with an increased risk of colorectal cancer and advanced prostate cancer (4–7). Several epidemiological studies have reported an association between salt-preserved foods, such as cured meats, and an increased risk of gastric cancer (8–10).

However, most previous studies on meat consumption and cancer have focused on incidence rather than mortality and have largely been conducted in Western populations. Evidence suggests that associations between meat consumption and cancer mortality may vary across populations, potentially reflecting climatic, environmental, and dietary heterogeneity (11). In addition, epidemiological findings indicate that cancer outcomes may differ by meat subtype, raising the possibility that such differences extend beyond cancer incidence to cancer mortality (12, 13). Although meat consumption has become increasingly prevalent in Asian countries, epidemiological evidence examining specific meat subtypes in relation to site-specific cancer mortality in Asian populations remains limited.

Therefore, our study aimed to investigate the associations between consumption of various meat types including red meat (beef and pork), processed meat, organ meat, and chicken and site-specific cancer mortality among Korean adults, providing evidence that may inform future public health strategies and targeted dietary interventions.

## 2 Materials and methods

### 2.1 Cohort construction and study population

The Korean Genome and Epidemiology Study (KoGES) is a large-scale prospective cohort study initiated by the Korea Centers for Disease Control and Prevention and the National Institute of Health in 2001 to investigate genetic and environmental determinants of major chronic diseases in the Korean population. KoGES comprises multiple cohort studies, including the community-based Korean Association Resources (KARE) cohort, the urban-based Health Examinees (HEXA) cohort, and the rural-based Cardiovascular Disease Association Study (CAVAS), among others. As of the end of 2014, approximately 245,000 participants had been enrolled at baseline, with comprehensive data collected through questionnaires, physical examinations, and biological specimen collection (14).

Our study population was derived from three population-based KoGES cohorts: KARE, CAVAS, and HEXA. The KARE cohort enrolled 10,030 participants aged 40–69 years from urban (Ansan) and rural (Anseong) areas between 2001 and 2002, with follow-up through 2014. The CAVAS cohort included 28,337 participants aged 40–69 years residing in rural areas of Korea, recruited between 2005 and 2011. The HEXA cohort comprised 173,195 urban residents aged ≥40 years who participated in health examinations between 2004 and 2013. These cohorts collected detailed demographic, socioeconomic, anthropometric, health-related, and dietary information.

**Figure 1:**
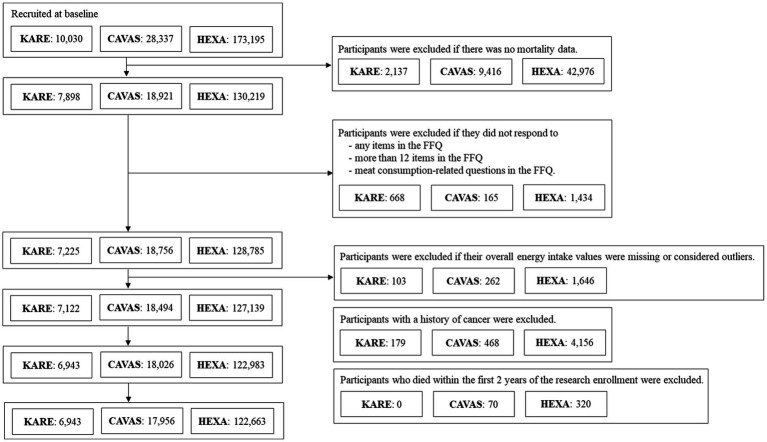
Flow chart of study participants.

For our study, anonymized data from 211,562 participants aged ≥40 years were linked to the death certificate database of the Korean National Statistical Office. Among these, 157,033 participants provided consent for linkage to the National Death Registry. Participants who did not complete the food frequency questionnaire, had missing or extreme energy intake values (defined as <mean −3 SD or >mean +3 SD after logarithmic transformation), had a history of cancer at baseline, or died within 2 years of cohort enrollment were excluded (Figure 1).

The KoGES data were fully anonymized, and the requirement for informed consent was waived by the Institutional Review Board of Seoul National University Hospital (IRB No. E-2208-063-1349).

### 2.2 Meat intake information

Participants’ meat and nutrient intake were assessed using a semi-quantitative food frequency questionnaire (SQFFQ) administered at baseline for each cohort (106 food items for the HEXA and CAVAS cohorts and 103 food items for the KARE cohort). The FFQ used in the KoGES is a semi-quantitative instrument that has been previously validated in the Korean population and is designed to assess usual dietary intake over the preceding year (15).

Food consumption frequency was categorized into nine levels, ranging from “never” to “three times or more per day,” and portion size was classified into three levels (one-half, one, and one and a half servings). Energy and macronutrient intakes were calculated using the food composition table developed by the Korean Rural Development Administration.

Meat intake was categorized into red meat (beef and pork), chicken, organ meat, processed meat (processed products such as ham, bacon and sausage, primarily derived from pork or red meat; processed poultry was not included), and total meat (the sum of red meat, chicken, organ meat, and processed meat) by extracting meat weight using standardized food recipe information.

### 2.3 Ascertainment of cancer mortality

Dates and causes of death were ascertained through linkage with the death certificate database of the Korean National Statistical Office from baseline through December 31, 2018.

Cancer-specific mortality was defined using the 10th revision of the International Classification of Diseases (ICD-10) codes C00–C97. To ensure statistical stability of site-specific analyses, cancer sites with fewer than 50 deaths in the combined KARE, CAVAS, and HEXA cohorts were excluded.

Accordingly, cancer types included in the analyses were lung (C34), liver (C22), colorectal (C18–C20), gastric (C16), pancreatic (C25), colon (C18), rectal (C19–C20), and prostate cancer (C61) in men, and lung (C34), pancreatic (C25), colorectal (C18–C20), liver (C22), colon (C18), breast (C50), gastric (C16), and ovarian cancer (C56) in women.

### 2.4 Statistical analysis

Participants were categorized into sex-specific quartiles according to total meat intake and intake of individual meat subtypes. Continuous variables are presented as means ± standard errors or as medians with interquartile ranges for non-normally distributed variables, and categorical variables are presented as frequencies and percentages. Hazard ratios (HRs) and 95% confidence intervals (CIs) for cancer-specific mortality were estimated using Cox proportional hazards models, with the lowest quartile (Q1) serving as the reference category.

To account for heterogeneity in baseline hazards, models were stratified by age group (<50, 50 to <60, and ≥60 years) and by study cohort (KARE, CAVAS, and HEXA). Models were further adjusted for age (continuous), body mass index (<23, 23 to <25, and ≥25 kg/m^2^), smoking (pack-years, continuous), alcohol intake (0, >0 to <5, 5 to <15, and ≥15 g/day), education level (elementary school or less, middle school, high school or more), physical activity (KARE: tertiles of MET-hours/week; CAVAS and HEXA: none, 1–4 times/week, ≥5 times/week), and total energy intake (continuous, kcal). Stratified analyses were additionally conducted according to age, body mass index, and smoking status. For total meat, red meat, chicken, and organ meat, HRs comparing the highest quartile with the lowest quartile were estimated for men, whereas HRs comparing the third quartile with the lowest quartile were estimated for women. Due to the small number of events in the highest quartile among women, comparisons were made between the third quartile and the lowest quartile to ensure statistical stability. For processed meat, HRs were estimated by comparing consumers with non-consumers for both sexes. All statistical analyses were performed using SAS version 9.4 (SAS Institute, Cary, NC, United States). Two-sided *p* values <0.05 were considered statistically significant.

## 3 Results

### 3.1 Baseline characteristics of study participants

Baseline demographic and nutritional characteristics of study participants across quartiles of total meat intake are presented in Table 1. A total of 147,562 participants from the KARE, CAVAS, and HEXA cohorts were included, of whom 53,847 (36.5%) were men and 93,715 (63.5%) were women. The mean age was 54.4 years (SD 9.04) for men and 53.2 years (SD 8.45) for women.

**Table 1:** Baseline characteristics of study participants by quartiles of total meat intake.

Characteristic	Men				Women			
	Q1	Q2	Q3	Q4	Q1	Q2	Q3	Q4
*N*	13,461	13,462	13,462	13,462	23,428	23,429	23,429	23,429
Study cohort, *n*(%)								
KARE	578 (4.3)	776 (5.8)	863 (6.4)	1,131 (8.4)	845 (3.6)	830 (3.5)	940 (4.0)	980 (4.2)
CAVAS	2,848 (21.3)	1,704 (12.7)	1,316 (9.8)	1,052 (7.8)	5,435 (23.2)	2,662 (11.4)	1,764 (7.5)	1,175 (5.0)
HEXA	10,035 (74.5)	10,982 (81.6)	11,283 (83.8)	11,279 (83.8)	17,148 (73.2)	19,937 (85.1)	20,725 (88.5)	21,274 (90.8)
Age (yrs)	58.3 ± 8.7	54.7 ± 8.7	52.9 ± 8.7	51.6 ± 8.6	57.3 ± 8.4	53.7 ± 8.0	51.6 ± 7.9	50.3 ± 7.8
BMI (kg/m^2^)	24.1 ± 2.8	24.3 ± 2.7	24.5 ± 2.8	24.7 ± 2.8	24.0 ± 3.1	23.8 ± 3.0	23.7 ± 3.0	23.6 ± 3.0
Smoking status, *n*(%)								
Never	4,143 (30.8)	3,793 (28.2)	3,550 (26.4)	3,248 (24.2)	22,549 (96.6)	22,595 (96.7)	22,485 (96.4)	22,305 (95.6)
Past	5,559 (41.4)	5,456 (40.6)	5,273 (39.2)	5,010 (37.3)	314 (1.3)	275 (1.2)	278 (1.2)	337 (1.4)
Current	3,738 (27.8)	4,190 (31.2)	4,621 (34.4)	5,180 (38.6)	484 (2.1)	490 (2.1)	569 (2.4)	697 (3.0)
Alcohol intake (g/d)	12.3 ± 32.1	14.9 ± 24.9	17.6 ± 28.2	22.7 ± 43.4	1.1 ± 5.7	1.6 ± 7.4	2.1 ± 8.6	3.0 ± 11.7
Education level, *n*(%)								
Less than middle school	2,997 (22.5)	1,725 (12.9)	1,348 (10.1)	1,135 (8.5)	9,968 (43.0)	5,882 (25.3)	4,257 (18.4)	3,236 (13.9)
Middle school	2,197 (16.5)	1,880 (14.1)	1,657 (12.4)	1,590 (11.9)	4,347 (18.7)	4,533 (19.5)	3,804 (16.4)	3,578 (15.4)
High school or more	8,133 (61.0)	9,725 (73.0)	10,338 (77.5)	10,633 (79.6)	8,894 (38.3)	12,827 (55.2)	15,127 (65.2)	16,407 (70.7)
Physical activity, *n*(%)								
Low	6,313 (50.0)	5,785 (46.7)	5,565 (45.5)	5,589 (47.0)	12,337 (55.6)	11,460 (51.7)	11,329 (51.7)	11,655 (53.5)
Middle	3,646 (28.8)	4,289 (34.6)	4,451 (36.4)	4,275 (35.9)	5,506 (24.8)	6,703 (30.3)	6,793 (31.0)	6,586 (30.3)
High	2,680 (21.2)	2,309 (18.7)	2,223 (18.1)	2,033 (17.1)	4,331 (19.6)	3,989 (18.0)	3,784 (17.3)	3,523 (16.2)
Menopausal status, *n*(%)								
Pre-menopause	N/A	N/A	N/A	N/A	5,079 (21.8)	8,291 (35.5)	10,613 (45.5)	12,214 (52.4)
Post-menopause	N/A	N/A	N/A	N/A	18,248 (78.2)	15,041 (64.5)	12,698 (54.5)	11,108 (47.6)
Energy intake (kcal/d)	1,550.5 ± 385.3	1,711.9 ± 398.7	1,885.9 ± 431.1	2,230.1 ± 547.4	1,426.0 ± 404.5	1,560.0 ± 416.7	1,713.9 ± 448.8	2,049.1 ± 557.1
Total carbohydrate intake (g/d)	298.0 ± 73.8	315.5 ± 76.3	332.2 ± 82.0	356.9 ± 91.7	277.0 ± 78.9	291.6 ± 80.8	307.5 ± 85.8	335.6 ± 94.0
Total protein intake (g/d)	45.5 ± 14.9	53.5 ± 14.4	63.4 ± 16.0	86.0 ± 25.7	42.3 ± 15.7	49.1 ± 15.7	57.4 ± 16.9	78.3 ± 26.3
Total fat intake (g/d)	16.9 ± 8.5	23.5 ± 8.6	31.3 ± 9.5	49.1 ± 17.8	14.5 ± 8.5	20.0 ± 8.8	20.5 ± 9.8	42.6 ± 18.2
Meat consumption (g/d)								
Total meat	14.2 (8.3, 19.2)	34.1 (28.9, 39.2)	58.9 (51.6, 68.4)	116.0 (94.6, 153.9)	7.0 (2.5, 10.8)	21.5 (17.7, 25.4)	41.0 (35.2, 48.2)	87.2 (69.5, 119.6)
Red meat	12.1 (6.7, 16.3)	29.1 (24.7, 33.8)	51.3 (44.7, 59.8)	103.5 (83.9, 137.1)	5.4 (1.7, 8.6)	18.2 (14.9, 21.5)	35.4 (30.3, 41.7)	77.4 (60.9, 107.5)
Beef	0 (0, 2.4)	6.6 (5.0, 8.2)	14.2 (12.2, 17.8)	39.3 (28.4, 57.6)	0 (0, 1.5)	5.0 (4.0, 6.5)	11.7 (9.2, 14.2)	33.7 (23.0, 51.5)
Pork	5.8 (2.5, 9.0)	16.1 (13.3, 19.2)	30.1 (26.7, 34.9)	67.2 (51.3, 82.0)	1.65 (0, 4.17)	10.00 (7.39, 11.67)	19.22 (16.46, 25.00)	46.70 (35.07, 67.60)
Chicken	0 (0, 0)	2.5 (2.5, 2.5)	3.8 (3.1, 3.8)	6.3 (6.3, 16.1)	0 (0, 0)	1.3 (1.3, 1.3)	2.5 (2.5, 2.5)	6.3 (6.3, 16.1)
Organ meat	0 (0, 0)	0.2 (0.2, 0.4)	1.7 (1.0, 1.8)	4.2 (2.3, 5.1)	0 (0, 0)	0.2 (0.2, 0.2)	0.9 (0.4, 1.7)	2.5 (1.8, 4.3)
Processed meat	0 (0, 0)	0.7 (0.7, 0.7)	1.3 (1.3, 1.3)	4.3 (3.3, 8.6)	0 (0, 0)	0.7 (0.7, 0.7)	1.3 (1.3, 1.7)	4.3 (3.3, 8.6)

Meat intake values are presented as median (25th, 75th percentile); other variables are expressed as *n* (%) or mean ± SD.

Among both men and women, participants in higher quartiles of total meat intake tended to be younger, highly educated, and more likely to smoke and consume alcohol. They were also less likely to engage in vigorous physical activity. Higher total meat intake was associated with a higher BMI among men, whereas a lower BMI was observed among women. Among women, higher meat intake was more frequently observed in the premenopausal group. Total energy intake increased across quartiles of total meat intake.

The median intake of total meat was 59.2 g/day for men and 43.1 g/day for women. Red meat accounted for nearly 90% of total meat intake, with pork constituting the predominant source of red meat in both sexes.

### 3.2 Association between meat consumption and cancer mortality

There were no significant associations observed between total meat intake and mortality from gastric, colorectal, colon, rectal, liver, pancreatic, or lung cancer in either men or women. Similarly, there were no significant associations observed for prostate cancer in men or for breast and ovarian cancer in women.

**Figure 2:**
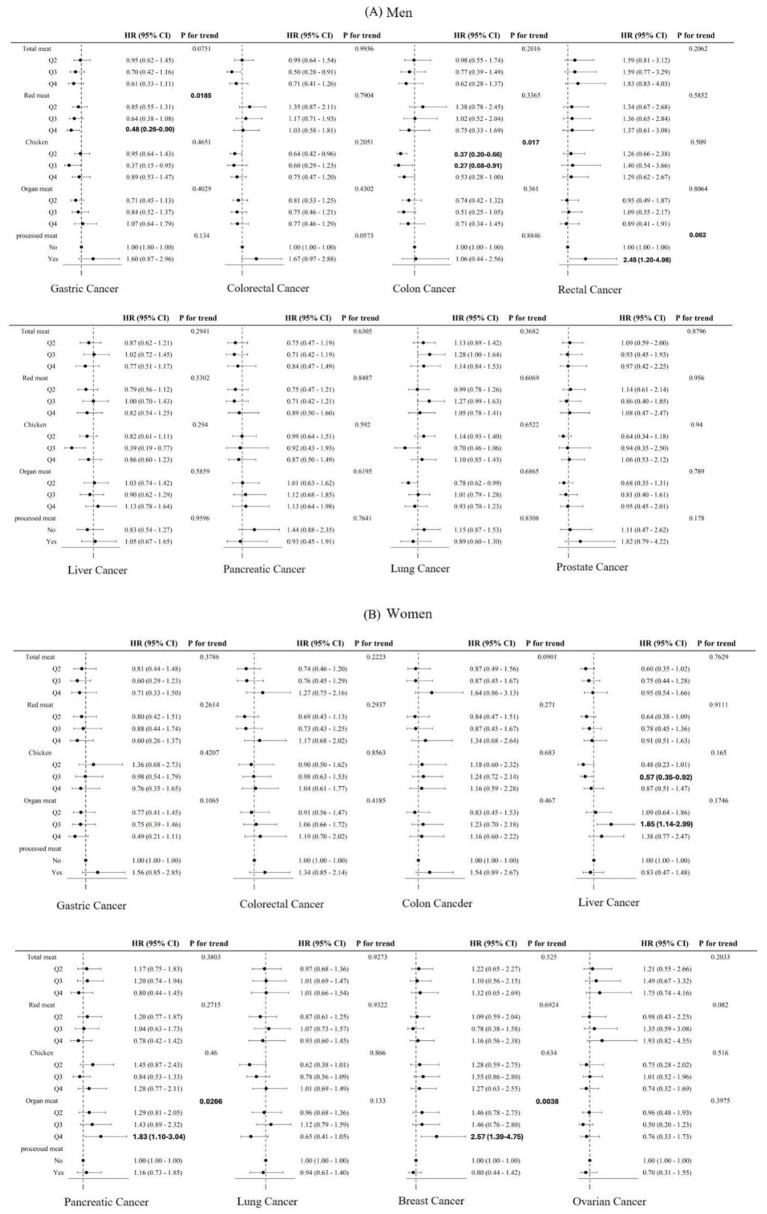
Association between meat consumption and site-specific cancer mortality by sex. **(A)** Men. **(B)** Women. Hazard ratios (HRs) and 95% confidence intervals (CIs) were estimated using Cox proportional hazards models stratified by age group (<50, 50 to <60, and ≥60 years) and study cohort (KARE, CAVAS, and HEXA). Models were adjusted for age (continuous), body mass index (<23, 23 to <25, ≥25 kg/m²), smoking (pack-years, continuous), alcohol intake (0, >0 to <5, 5 to <15, ≥15 g/day), education level (elementary school or less, middle school, high school or more), physical activity (KARE: tertiles of MET-hours/week; CAVAS and HEXA: none, 1–4 times/week, ≥5 times/week), and total energy intake (continuous, kcal). Meat intake was categorized into sex-specific quartiles. Processed meat was analyzed as consumers versus non-consumers. The lowest quartile (Q1) served as the reference category. For red meat and organ meat, additional adjustment was made for chicken and processed meat intake; for processed meat and chicken, models were further adjusted for other meat subtypes.

In men, higher red meat intake was inversely associated with gastric cancer mortality (HR 0.48, 95% CI 0.26–0.90; *p* for trend = 0.0185), and higher chicken intake was associated with a lower risk of colon cancer mortality (HR 0.53, 95% CI 0.28–1.00; *p* for trend = 0.017). In contrast, higher processed meat intake was associated with increased rectal cancer mortality (HR 2.45, 95% CI 1.20–4.98; *p* for trend = 0.0062) (Figure 2A). In sensitivity analyses separating red meat into beef and pork, inverse associations with gastric cancer mortality were observed for both beef and pork, although statistical significance was observed only for beef (Q4 vs. Q1: HR 0.37, 95% CI 0.18–0.74; *p* for trend = 0.0027), while pork showed a similar inverse trend (Q4 vs. Q1: HR 0.64, 95% CI 0.36–1.16; *p* for trend = 0.0854) (Supplementary Table 1A).

In women, higher organ meat intake was associated with increased pancreatic cancer mortality (HR 1.83, 95% CI 1.10–3.04; *p* for trend = 0.0266) and increased breast cancer mortality (HR 2.57, 95% CI 1.39–4.75; *p* for trend = 0.004) (Figure 2B).

### 3.3 Subgroup analyses by age, BMI, and smoking status

**Figure 3:**
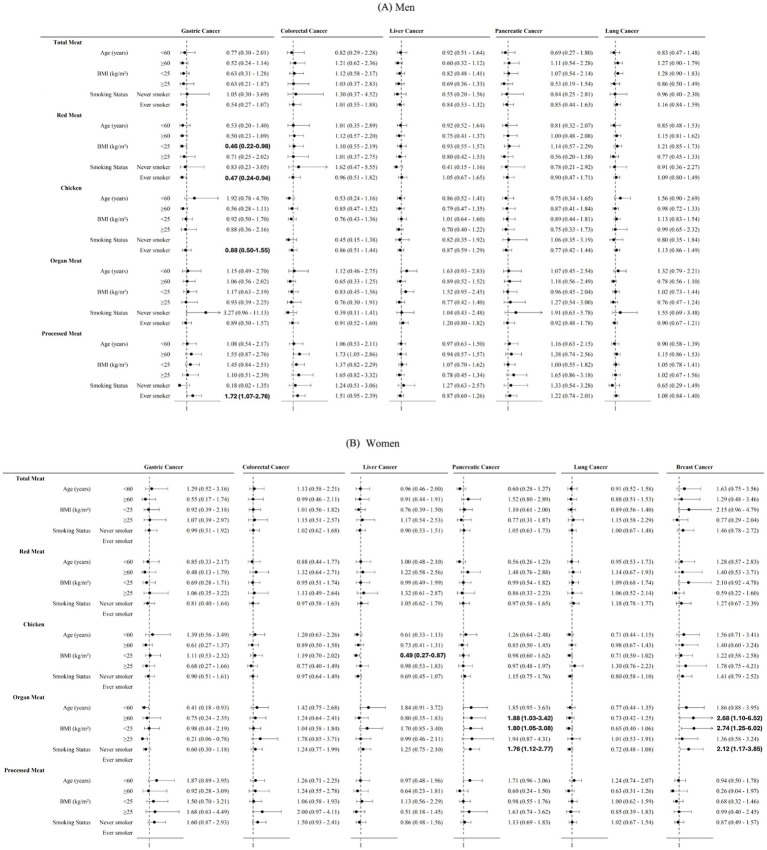
Subgroup analyses of the association between meat consumption and site-specific cancer mortality by age, body mass index, and smoking status. **(A)** Men. **(B)** Women. Subgroup analyses were performed according to age, body mass index, and smoking status using multivariable-adjusted Cox proportional hazards models. Meat intake was categorized into sex-specific quartiles; processed meat was analyzed as consumers versus non-consumers. The lowest quartile (Q1) served as the reference category. Models were adjusted for the same covariates as in Figure 2.

In men, subgroup analyses showed significant associations primarily for gastric cancer. There were no significant associations observed in age-stratified analyses. Among participants with a BMI < 25 kg/m^2^, higher red meat intake was associated with lower gastric cancer mortality (HR 0.46, 95% CI 0.22–0.98). Among ever smokers, higher red meat intake was inversely associated with gastric cancer mortality (HR 0.47, 95% CI 0.24–0.94), whereas higher processed meat intake was associated with increased gastric cancer mortality (HR 1.72, 95% CI 1.07–2.76) (Figure 3A).

In women aged ≥60 years, higher organ meat intake was associated with increased mortality from pancreatic cancer (HR 1.88, 95% CI 1.03–3.42) and breast cancer (HR 2.68, 95% CI 1.10–6.52). Among participants with a BMI < 25 kg/m^2^, higher chicken intake was associated with lower liver cancer mortality (HR 0.49, 95% CI 0.27–0.87), whereas higher organ meat intake was associated with increased pancreatic and breast cancer mortality. Similar associations between organ meat intake and increased pancreatic and breast cancer mortality were observed among never smokers (Figure 3B).

## 4 Discussion

In this study, we investigated the association between meat consumption and site-specific cancer mortality among Korean adults. Overall, there was no statistically significant association between total meat consumption and cancer mortality. In men, higher red meat consumption was associated with lower gastric cancer mortality, whereas higher processed meat consumption was associated with increased rectal cancer mortality. In women, higher organ meat consumption was associated with increased mortality from pancreatic and breast cancer.

### 4.1 Total meat consumption and cancer mortality

Previous studies conducted predominantly in Western populations have reported positive associations between meat consumption and cancer mortality, particularly for red and processed meat (11, 16–18). Several biological mechanisms have been proposed, including exposure to heterocyclic amines and polycyclic aromatic hydrocarbons generated during high-temperature cooking, as well as heme iron–related lipid peroxidation and formation of N-nitroso compounds (19–23). More recently, interactions between dietary Neu5Gc and endogenous inflammatory responses have also been suggested (24, 25).

However, evidence from Asian populations has been less consistent, with several studies reporting null associations between total meat intake and cancer mortality or even inverse associations for certain outcomes (26–28). Consistent with these findings, our study did not observe an association between total meat intake and overall cancer mortality.

These discrepancies may partly reflect regional differences in dietary patterns. Although red meat, predominantly pork, accounted for the majority of total meat intake in our study population, overall meat consumption in Korea remains lower than the OECD average (29). In addition, Asian dietary patterns are generally characterized by higher consumption of fish and other non-red meat protein sources, which have been suggested to confer potential protective effects against cancer (11, 26, 30). Together with rapid changes in obesity prevalence, physical activity, and access to medical care, these factors may influence baseline cancer mortality risk and attenuate the relative contribution of total meat intake through statistical interaction with other risk factors (16, 27).

### 4.2 Inverse association between red meat intake and gastric cancer mortality in men

Our study showed that higher red meat intake was inversely associated with gastric cancer mortality in men, particularly among individuals with lower BMI and among smokers in stratified analyses. This finding contrasts with reports from Western populations, where higher red meat consumption has been associated with increased gastric cancer incidence.

However, pooled analyses of Asian prospective cohort studies have not shown a significant association between red meat intake and cancer mortality, including gastric cancer mortality (27). Similarly, the Korea Health Examinees–GEM study reported no significant association between total red meat consumption and overall cancer mortality (31). In addition, a large meta-analysis from the Stomach Cancer Pooling (StoP) Project demonstrated that while red meat intake was associated with an increased risk of gastric cancer incidence in Europe and the United States, no statistically significant association was observed in Asian populations (32). Several factors may contribute to the inverse association observed in this study. First, this finding may reflect characteristics observed in Asian populations, in which higher meat consumption often accompanies relative social affluence. Evidence from a large Japanese prospective cohort suggests that, unlike in Western populations, higher red meat consumption was associated with greater intake of vegetables and fish and a lower intake of salt-preserved foods, resulting in a more favorable overall dietary balance (16, 26). Such overall nutritional balance may modify associations traditionally attributed to red meat consumption. In our sensitivity analysis, both beef and pork consumption showed a similar inverse trend with gastric cancer mortality, although statistical significance was observed only for beef. These findings suggest that the observed association is unlikely to be explained by a specific type of red meat, but may instead reflect broader dietary patterns and underlying socioeconomic factors associated with red meat consumption. Second, differences in meat type and preparation methods in Korea may also contribute to the observed association. Pork constitutes the predominant source of red meat and is more commonly prepared by grilling or roasting rather than curing or salting, as is typical of many processed meats in Western countries. These differences may result in lower salt exposure and a different fat composition compared with Western dietary patterns (33). Third, both Korea and Japan have maintained nationwide population-based gastric cancer screening programs for several decades, leading to substantial reductions in gastric cancer mortality through early detection and timely treatment (34). Individuals with higher socioeconomic status, who also tend to consume greater amounts of meat, may be more likely to undergo regular screening and have improved access to healthcare services, potentially contributing to the observed reduction in gastric cancer mortality.

### 4.3 Organ meat consumption and pancreatic and breast cancer mortality in women

Epidemiological evidence regarding meat consumption and pancreatic and breast cancer mortality in women remains inconsistent, as most prior studies have focused on red and processed meat and primarily examined cancer incidence rather than mortality (13, 35, 36). The Women’s Health Initiative study found no significant association between total protein intake and breast cancer incidence or mortality; although higher animal protein intake was associated with increased breast cancer incidence, protein intake was assessed as a broad exposure category, and specific meat subtypes such as organ meat were not evaluated separately (37).

In this study, higher consumption of organ meat was associated with increased pancreatic and breast cancer mortality among women; these associations were more pronounced in stratified analyses among women with lower BMI, older age, and non-smokers, whereas no comparable associations were observed for red or processed meat.

These findings suggest that organ meat may represent a distinct dietary exposure with potential relevance to cancer mortality in women. One possible explanation is higher internal exposure to toxic metals, as organ meats such as liver and kidney are known to contain substantially higher concentrations of arsenic, cadmium, and lead than skeletal muscle (38). These metals are detectable in adipose tissue, which has been proposed as a biologically relevant target of metal toxicity (39). Although much of the epidemiologic evidence derives from male-dominated cohorts, arsenic and cadmium exposure has been associated with pancreatic cancer risk and mortality, as well as breast cancer risk in women (40, 41). Given that women generally have a higher proportion of total body adiposity than men, with sex-specific fat distribution patterns, such characteristics may influence the storage and mobilization of toxic metals (42, 43). These stored compounds can be released into the circulation during periods of weight loss or age-related changes in fat distribution, leading to increased internal exposure despite similar dietary intake. Evidence indicates that persistent organic pollutants (POPs), which accumulate in adipose tissue, exhibit increased circulating concentrations following weight reduction, suggesting enhanced bioavailable exposure as adipose mass decreases (44). Such alterations in adipose tissue dynamics may amplify the biological effects of chronic low-level exposure to lipophilic pollutants and toxic metals.

Effect modification by smoking status is also biologically plausible. Tobacco smoke particulate contains metals such as cadmium and lead (45), and therefore dietary sources may account for a larger fraction of total internal metal burden among never-smokers, potentially strengthening diet–cancer associations in this group.

### 4.4 Strengths and limitations of the study

The strength of our study first lies in the use of data from a large-scale, population-based cohort in South Korea, enhancing the generalizability of the findings. To the best of our knowledge, this is the first Korean study to examine the associations between different meat subtypes and cancer-specific mortality. Whereas most previous studies have focused on overall cancer mortality in relation to total meat consumption, our study evaluated cause-specific cancer mortality according to meat subtype within a Korean population, thereby providing a novel and more detailed perspective on this topic.

However, several limitations should be acknowledged. First, heterogeneity across study populations may exist because participants were drawn from multiple cohorts. However, the majority of participants were derived from the HEXA cohort, and sensitivity analyses restricted to the HEXA population yielded results consistent with the overall findings (Supplementary Table 3). In addition, inclusion of both urban and rural populations may better reflect dietary patterns in the general Korean population. Second, although menopausal status was adjusted for in the analyses of women, we did not perform stratified analyses by menopausal status due to the limited number of events in each subgroup, which may have reduced statistical power and the stability of the estimates. Third, dose–response relationships were not evaluated using flexible modeling approaches, such as restricted cubic splines, due to the limited number of events. Further studies are needed to explore potential non-linear associations. Fourth, we were unable to account for changes in dietary habits over time, cooking methods, or cancer stage at diagnosis, all of which may influence cancer outcomes. Finally, residual or unmeasured confounding cannot be fully excluded despite adjustment for multiple covariates. Dietary intake was assessed using self-reported questionnaires, which are subject to measurement error and may have biased the results toward the null.

## 5 Conclusion

In this large population-based cohort study, total meat consumption was not associated with overall cancer mortality among Korean adults. However, sex-specific associations according to meat subtype were observed, with inverse associations between red meat intake and gastric cancer mortality in men and positive associations between organ meat intake and breast and pancreatic cancer mortality in women.

## Data Availability

The data analyzed in this study is subject to the following licenses/restrictions: the KoGES data are available upon reasonable request and with approval from the Korea Disease Control and Prevention Agency (KDCA), subject to data access regulations and ethical review. Data access requests can be submitted through the official KoGES website: https://www.kdca.go.kr.
